# Spin Funneling for Enhanced Spin Injection into Ferromagnets

**DOI:** 10.1038/srep28868

**Published:** 2016-07-04

**Authors:** Shehrin Sayed, Vinh Q. Diep, Kerem Yunus Camsari, Supriyo Datta

**Affiliations:** 1 School of Electrical and Computer Engineering, Purdue University, West Lafayette, IN 47907, USA

## Abstract

It is well-established that high spin-orbit coupling (SOC) materials convert a charge current density into a spin current density which can be used to switch a magnet efficiently and there is increasing interest in identifying materials with large spin Hall angle for lower switching current. Using experimentally benchmarked models, we show that composite structures can be designed using existing spin Hall materials such that the effective spin Hall angle is larger by an order of magnitude. The basic idea is to funnel spins from a large area of spin Hall material into a small area of ferromagnet using a normal metal with large spin diffusion length and low resistivity like Cu or Al. We show that this approach is increasingly effective as magnets get smaller. We avoid unwanted charge current shunting by the low resistive NM layer utilizing the newly discovered phenomenon of pure spin conduction in ferromagnetic insulators via magnon diffusion. We provide a spin circuit model for magnon diffusion in FMI that is benchmarked against recent experiments and theory.

## Introduction

**Figure 1:**
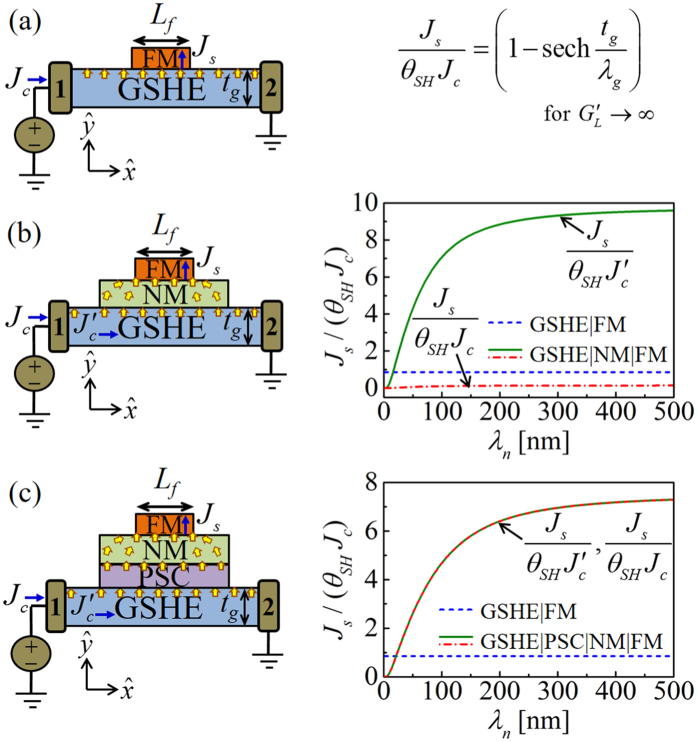
(**a**) Schematic structure of giant spin Hall effect (GSHE) metal driving a ferromagnet (FM), governed by Eq. (1). In this structure, *J*_*s*_/(*θ*_*SH*_*J*_*c*_) ≤ 1. (**b**) An intermediate normal metal (NM) layer with low resistivity and large spin diffusion length will funnel spins from large area of the GSHE into the small area of FM, making 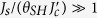 in terms of charge current density in GSHE 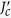 (green curve). Current shunting by low resistive NM layer will cause 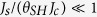 in terms of total charge current density (*J*_*c*_) in the structure (red curve), as there is a large component of the charge current outside GSHE which does not generate spin currents. (**c**) Current shunting effect can be avoided by utilizing pure spin conduction (PSC) via magnon diffusion in thin ferromagnetic insulator. The composite GSHE|PSC|NM provide larger effective spin Hall angle i.e. 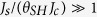 in terms of total charge current density in the structure. Note that enhancement is slightly reduced compared to structure in (**b**) due to insertion of the PSC layer. In simulation, Cu, YIG, and W parameters are used as NM, PSC, and GSHE layers while FM is assumed to be a perfect spin sink 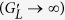 in this figure. GSHE, PSC, and NM lengths are 700, 500, and 500 nm respectively and thicknesses are 6, 4, and 20 nm respectively. Length of FM is 10 nm.

Magnetization switching with high spin-orbit coupling (SOC) materials such as the giant spin Hall effect (GSHE) metals^1^,^2^,^3^,^4^,^5^,^6^,^7^,^8^ and topological insulator surface states^9^ have attracted much attention for potential memory^10^,^11^ and logic^12^,^13^ device applications. In these materials (see Fig. 1(a)), a longitudinal charge current density (*J*_*c*_) induces a transverse spin current density which if large enough can switch a ferromagnet (FM)^14^,^15^. The ratio of spin current density (*J*_*s*_) injected into a spin load to *θ*_*SH*_*J*_*c*_ is given by


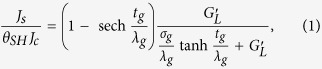


where *t*_*g*_, *λ*_*g*_, *σ*_*g*_, and *θ*_*SH*_ are thickness, spin diffusion length, conductivity, and intrinsic spin Hall angle of GSHE. 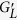 is the spin conductance per unit area of the spin load (see Supplementary Information for derivation). The right-hand side consists of two factors each of which has a maximum value of one i.e. *J*_*s*_/(*θ*_*SH*_*J*_*c*_) ≤ 1. The first term has been noted previously^16^, which represents the cancellation from oppositely spin polarized back surface. This term can be maximized by using thick layers (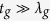) while the second term is maximized if 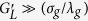. Even under optimal conditions the maximum spin current density is limited by *θ*_*SH*_*J*_*c*_, and there is a major research effort on finding materials with increased spin Hall angle *θ*_*SH*_^1^,^2^,^3^,^4^.

This paper proposes a different approach based on existing materials that are already being used. Using experimentally benchmarked models we show that composite structures designed with existing spin Hall materials could lead to an order large “effective” spin Hall angle i.e. 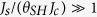. The method proposed here is increasingly effective as magnets get smaller, making our approach particularly useful for small magnets which present a formidable challenge because of the high spin current density requirement for switching^17^. We believe this method could be useful in future device designs, irrespective of the detailed mechanisms underlying the spin-orbit interaction which is a question of active research and debate^1^,^3^,^18^,^19^. For quantitative evaluation of the proposal, we rely on our spin circuit model for GSHE^20^ which was derived based on the widely used semiclassical theory of spin Hall effect^21^. We recognize that the actual circuit parameters may change as we explore new materials and our understanding evolves, but we believe the structure of the circuit is quite generic since different mechanisms of generating various spin-orbit torques result in similar terminal characteristics^18^ in the circuit.

### Spin Funneling

The basic idea is simply to funnel spins from a large area of the GSHE material into a small area of the magnet using an intermediate normal metal (NM) layer with large spin diffusion length (*λ*_*n*_) and low resistivity (e.g. copper, aluminum etc.), as shown in Fig. 1(b). At best one might expect an increase in *J*_*s*_ by a factor equal to the ratio of the length of the NM layer (*L*_*n*_) to the length of the FM layer (*L*_*f*_), which in our simulation is ∼50. In practice we expect the improvement to be much smaller because of spin loss if *L*_*n*_ > *λ*_*n*_ and the additional resistance of the funnel layer. Our 2D simulation predicts a more modest increase in *J*_*s*_ by a factor of ∼10, which is still quite significant.

### Current Shunting Effect

The structure in Fig. 1(b), however, provides an increase in *J*_*s*_ relative to 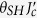 (green curve in Fig. 1(b)) where 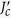 is the charge current density flowing in the GSHE material and shows decrease in *J*_*s*_ relative to *θ*_*SH*_*J*_*c*_ (red curve in Fig. 1(b)) where *J*_*c*_ is the total charge current density flowing in from the terminals. This is because the NM layer needed to funnel the spin current also provides a shunt path to the charge current, and there is a large component of the charge current outside the GSHE which does not generate spin currents.

### Pure Spin Conductor

We argue that this type of problem^22^ can be overcome by using an important new discovery namely that of pure spin conduction in ferromagnetic insulators (FMI) like yttrium-iron-granet (YIG) which do not allow charge currents to flow, but nevertheless allow longitudinal spin currents to flow through magnon generation^23^,^24^,^25^,^26^,^27^,^28^,^29^,^30^. Such pure spin conductors are described by a conductance matrix of the following form


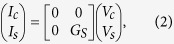


where *I*_*c*_, *I*_*s*_ are terminal charge, spin currents and *V*_*c*_, *V*_*s*_ are terminal charge, spin voltages. Based on available experimental data^27^,^28^,^29^,^30^ we have developed a spin circuit model for such pure spin conductors (PSC), which we use in conjunction with existing models for GSHE, NM, and FM layers to obtain the result in Fig. 1(c) showing an increase in *J*_*s*_ by a factor of ~7, which is less than that in Fig. 1(b), but still quite significant. A very thin layer of FMI is required to have good spin transmission, which seems feasible as few nanometer thick YIG layers have been fabricated^31^. The spin injection and transmission through FMI may be limited by large interface resistances^24^,^32^,^33^, which we model separately with interface modules.

### Effective Spin Hall Angle

Note that in Fig. 1(c), the increase is with respect to the total charge current density (*J*_*c*_), so that one could view the composite GSHE|PSC|NM as an effective material whose “effective” spin Hall angle is ∼7 times larger than that of the GSHE. This represents a significant increase and even larger increases may be possible with two dimensional funneling: our simulation assumes constant width in the third dimension (perpendicular to the paper) so that the funneling occurs only in one dimension. Two dimensional funneling structures are more difficult to analyze, but should be no more difficult to fabricate.

### Spin Load Characteristics

We should emphasize the importance of the spin load conductance 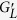 in limiting the degree of funneling achievable in practice through the second factor in Eq. (1). Usually the FM has a very high spin conductance, so that 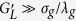. By making the effective GSHE area (*A*_*g*_) larger than the FM area (*A*_*f*_), we make *σ*_*g*_*A*_*g*_/*λ*_*g*_ larger and if it becomes comparable to 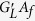, the second factor in Eq. (1) will adversley affect spin injection. Consequently, spin funneling is a technique to enhance spin injection into low spin resistive load 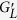 like FM but may not enhance spin injection into high spin resistive loads like semiconductors.

### Prior Experiments

Prior efforts of incorporating NM layer between GSHE and FM in spin pumping experiments have reported increased effective spin mixing conductance^34^,^35^, lowering of effective Gilbert damping^35^,^36^,^37^, and predicted lower switching current density^37^. It was experimentally shown that the effect of a thin intermediate copper layer is same on spin pumping induced inverse spin Hall effect and damping-like torque driven by spin Hall effect^36^. Gilbert damping and switching current was reduced by a factor of ~2 experimentally by insertion of thin layer of hafnium between CoFeB and Pt^38^. An intermediate Cu layer was used between GSHE and FM to quantify the contributions from bulk and interface spin-orbit coupling^39^. These efforts focused on changing the interface properties between GSHE and FM. In this work, we discuss the effect of spin funneling caused by 2D spin diffusion in the bulk of the intermediate NM layer, which we believe is different from the interface effects reported previously.

### Outline

The outline of this paper is as follows. We start with a description of the spin circuit models that we use for simulation, followed by a description of the spin circuit model for the newly discovered pure spin conductors (PSC) which has not been discussed before. We then present numerical results showing how the funneling effect is influenced by the resistivity (*ρ*_*n*_) and spin coherence length (*λ*_*n*_) of the NM layer along with its length (*L*_*n*_) and thickness (*t*_*n*_), FM conductance (*G*_*L*′_), magnon resistivity (*ρ*_*m*_) of the PSC layer along with its thickness (*d*), and the interface resistance between PSC and adjacent layers. We end with a brief summary.

## Model

**Figure 2:**
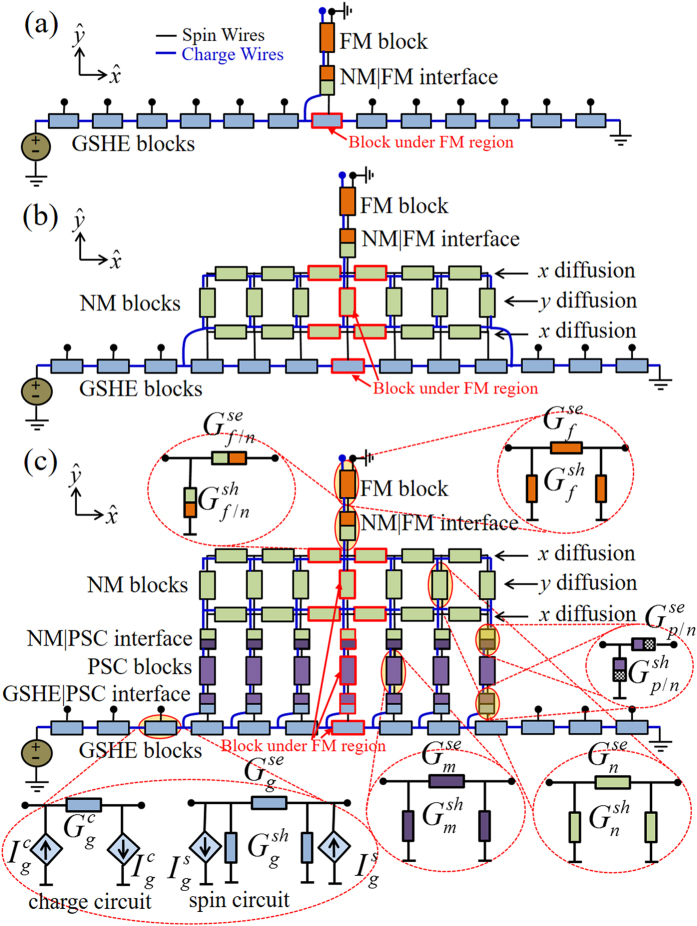
Simulation models using experimentally benchmarked multi-physics framework^40^. We use four different spin circuit models: GSHE, NM, FM, and PSC to construct distributed circuits using standard circuit technique and represent structures in (**a**) Fig. 1(a), (**b**) Fig. 1(b), and (**c**) Fig. 1(c), and analyzed with standard solvers like SPICE. The FMI interface with adjacent layers were taken into account with interface modules. Red shaded blocks represent the region right under the FM. FM load is modeled with bulk FM module and FM|NM interface module. The boundary condition of the other end of FM is kept spin grounded and charge open. The NM layer is modeled as a ladder structure with two longitudinal NM block rows taking into account the spin diffusion along 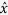-direction and the transverse NM blocks connecting two longitudinal rows take into account the spin diffusion along 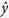-direction. In the simulation, GSHE length is greater than the NM or PSC length and the lengths of NM and PSC are kept equal.

We have performed 2D simulations using our experimentally benchmarked multi-physics spin circuit framework^40^ which can be viewed as an extension of the earlier works^41^,^42^,^43^ on the spin circuit theory of NM|FM structures to include a wide variety of materials and phenomena. For the structures in Fig. 1, we use spin circuit models for four distinct materials of which three (NM, FM, and GSHE) are available from prior works^20^,^41^,^42^,^43^. For the convenience of the reader, detailed description of the models are provided as the Supplementary Information. For the fourth material (PSC) we develop a new spin circuit model in the next section and compare it with recent experiments as well as standard theory. The interfaces between PSC and adjacent layers are treated by modifying the interface conductance matrix of FMI|NM^20^,^21^,^44^ to incorporate the conductance for spins that are collinear to the magnetization direction^24^,^32^,^33^. We use these four types of modules to construct distributed circuits using standard circuit rules (see Fig. 2(a–c)) to represent the structures in Fig. 1(a–c) respectively and analyzed with standard solvers like SPICE.

### Structure

The length of the GSHE layer (*L*_*g*_) is larger than the length of NM and PSC layers which is kept fixed at 700 nm in all of our simulations. NM and PSC layers have equal lengths (*L*_*n*_) and much greater than the length of the FM layer (*L*_*f*_). We have varied the FM length from 5 to 20 nm and NM length from 100 to 500 nm. The thickness of GSHE (*t*_*g*_) is kept fixed at 6 nm for every simulation. The thicknesses of NM and PSC are 20 nm and 4 nm respectively, while we varied the NM thickness (*t*_*n*_) from 1 to 250 nm to observe the thickness dependence of spin funneling. Material parameters used for simulations are provided as the Supplementary Information.

### GSHE Layer

The length of each small GSHE block is set to 1 nm (less than the spin diffusion length of GSHE), except the red shaded block which represent the region right under the ferromagnet (see Fig. 2(a–c)). The length of the red shaded GSHE block is same as the length of FM. This block is directly connected to FM modules (bulk and interface) in Fig. 2(a) to construct the structure in Fig. 1(a). The thickness of all GSHE blocks are same and kept fixed at 6 nm. The circuit representation of GSHE have two parts: charge circuit for charge transport along 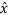-direction and spin circuit for spin transport along 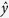-direction. The series conductance (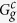) of charge circuit represents ordinary charge conductance of GSHE and current sources (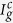) represent inverse spin Hall effect (ISHE). In spin circuit, series (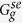) and shunt (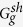) conductances are 3 × 3 matrices for three polarizations of spins (*z*, *x*, and *y*) and represent spin transmission and spin relaxation respectively along the 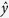-direction. The current sources 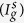 in spin circuit represent the spin Hall effect (SHE). Tungsten and platinum parameters have been used for simulation.

### NM Layer

We have discretized and modeled the NM layer in a ladder structure (see Fig. 2(b,c)) to capture the 2D spin diffusion. We have two longitudinal rows to capture spin diffusion along 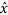-direction and one transverse column to capture spin diffusion along 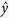-direction. The bottom row collects spins from GSHE blocks (directly in Fig. 2(b) and via PSC in Fig. 2(c)) and each NM block is connected between the spin terminals of two adjacent GSHE blocks. The length of each longitudinal NM block is the half of the summation of the two adjacent GSHE block lengths. Thus the length of each black and red shaded longitudinal blocks are 1 nm and (*L*_*f*_ + 1 nm)/2, respectively. The top row is same as the bottom row and it is connected to the FM modules. The thickness of each longitudinal block is half of the thickness of NM layer (*t*_*n*_/2), thus two longitudinal rows together captures the total thickness of the layer. The length of all transverse blocks is the thickness of NM layer (*t*_*n*_) and the thicknesses of transverse NM blocks is the length of the corresponding GSHE block (in Fig. 2(b)) or PSC block (in Fig. 2(c)), right under it. To take into account the current shunting in Fig. 2(b), the charge terminals of the left most and right most NM blocks in the bottom row are connected to the charge terminals of the adjacent GSHE blocks. In Fig. 2(c), charge and spin terminals of bottom row are connected to the charge and spin terminals of PSC blocks. Each NM block is a 4-component (1 charge and 3 spins) *π*-circuit with 4 × 4 series (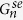) and shunt (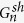) conductance matrices. Copper, aluminum, silver, and gold parameters have been used for simulation.

### PSC Layer

Each of the PSC blocks are connected (both charge and spin terminals) between bottom row of NM blocks and GSHE blocks along with interface blocks on both sides (see Fig. 2(c)). PSC blocks capture the spin transmission via magnon diffusion along 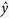-direction and spins that are collinear to the magnetization direction of the FMI are transmitted. The length of each block is the thickness of the FMI layer and the thickness of each block is the length of corresponding GSHE block right under it. Each block is a *π*-circuit with series (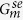) and shunt (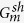) conductances representing magnon assisted spin transmission and relaxation respectively. The interface model has a series conductance (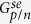) and a shunt conductance (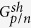). The series conductance captures the interface spin conductance for spins collinear to the magnetization direction while the shunt conductance captures the absorption of spins at the interface which are orthogonal to the magnetization direction. The models will be discussed in detail in the next section. Yttrium-iron-garnet parameters have been used for simulation.

### FM Layer

The bulk FM module is connected to the red shaded GSHE block (Fig. 2(a)) or red shaded NM blocks (Fig. 2(b,c)) via FM|NM interface module. In Fig. 2(a), the charge terminal of the the FM|NM module is attached to the left charge terminal of the red shaded GSHE block. The other end of the bulk FM module is kept spin grounded and charge open. We either assume the FM as perfect “spin sink” or set the magnetization direction of the FM perpendicular to the spin polarization direction in GSHE to observe the maximum spin current absorbed by the FM. This allows us to understand the increase in effective spin Hall angle. The damping-like and field-like torques are proportional to the effective spin Hall angle and depend on the real and imaginary parts of the interface spin mixing conductance incorporated in our FM|NM interface module^18^,^36^,^45^. To simulate the “spin sink”, we have applied ground boundary condition at the spin terminal of the block representing the region under the FM, instead of attaching FM and FM|NM interface modules. Otherwise, CoFeB, Co, and Py parameters have been used for simulation.

## Spin circuit model for magnon diffusion in FMI

**Figure 3:**
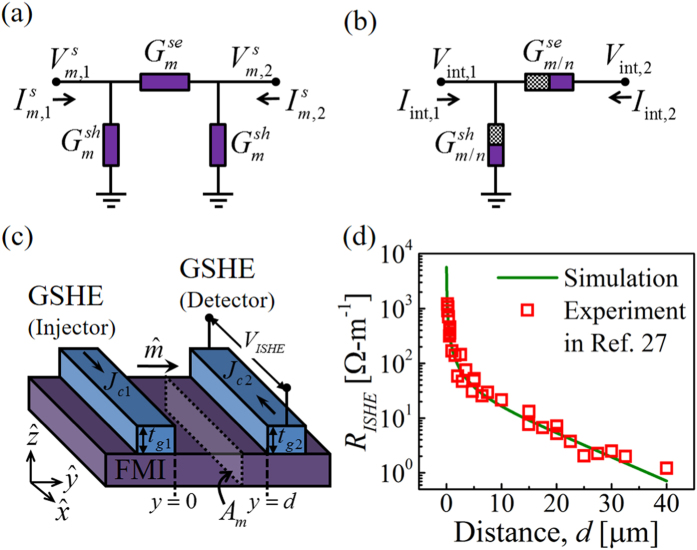
(**a**) Spin circuit representation for spin transport via magnon diffusion in the bulk of ferromagnetic insulators (FMIs). Series conductance represents transmission of spins through FMI layer and shunt conductances represent the spin lost due to magnon relaxation. (**b**) Spin circuit representation of FMI|NM interface. The series conductance consists of the interface conductance for spins collinear to the magnetization direction and shunt conductance consists of the real and imaginary parts of the interface spin mixing conductance for spins transverse to the magnetization direction. (**c**) Schematic structure of a GSHE|FMI|GSHE structure where left GSHE injects spins by spin Hall effect, which are transmitted via magnon diffusion in FMI, and detected at the right GSHE by inverse spin Hall effect. **(d**) Inverse spin Hall voltage per unit length at the detector GSHE to charge current flowing in the injector GSHE ratio (*R*_*ISHE*_) as a function of the distance between two GSHE contacts. We have compared our spin circuit results for Pt|YIG|Pt with the experiments in ref. 27. Comparison estimates the spin circuit parameters as: *ρ*_*m*_ = 10 *μ*Ω-cm, *λ*_*m*_ = 10 *μ*m, and *g*_*s*_ = 3.5 × 10^14^ S/m^2^.

Pure spin transport through FMI has been explored theoretically^23^,^24^,^25^,^46^,^47^ and observed experimentally^26^,^27^,^28^,^29^,^30^ in the past. In this section, we provide spin circuit representation for pure spin conduction (PSC) via magnon diffusion in the bulk of ferromagnetic insulators (FMI) such as yttrium-iron-garnet (YIG) (see Fig. 3(a)). We have also modified the FMI|NM interface model^20^,^21^,^44^ to take into account the interface conductance for spins that are collinear to the magnetization direction^24^,^32^,^33^ (see Fig. 3(b)).

### Spin Circuit Parameters

The spins collinear to the magnetization direction of FMI (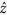-direction) will be transmitted via magnons. Both up and down spins will be transmitted as long as the non-equilibrium spin voltage applied at the FMI surface is much less than *k*_*B*_*T*/*q*, where *k*_*B*_ is the Boltzmann constant, *q* is the electron charge, and *T* is the absolute temperature. In our spin circuit model for magnon diffusion in the bulk of FMI, the series spin conductance 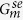 captures magnon assisted transmission of the injected spins and 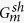 captures the loss of injected spins due to the magnon relaxation. In the charge (*c*) and *z*, *x*, *y* spin polarization basis, they can be written as


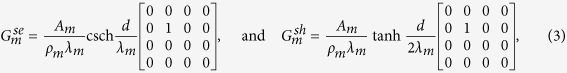


where *ρ*_*m*_ is the pure spin resistivity of the ferromagnetic insulator, *λ*_*m*_ is the magnon diffusion length, *d* is the thickness of FMI, and *A*_*m*_ is the cross-sectional area. The FMI|NM interface model consists of a series conductance (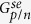) and a shunt conductance (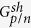). 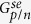 includes the interface conductance (*g*_*s*_) for the spins that are collinear to the magnetization direction while 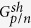 captures the spin absorption at the interface which are orthogonal to the magnetization direction. In the charge (*c*) and *z*, *x*, *y* spin polarization basis, they can be written as


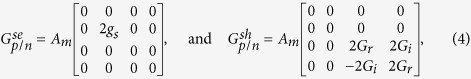


where *G*_*r*_ and *G*_*i*_ are the real and imaginary parts of interface spin mixing conductance. Note that *g*_*s*_, *G*_*r*_, and *G*_*i*_ are in the units of S/m^2^.

### Comparison with Experiment

In order to estimate our bulk and interface spin circuit parameters, we have considered a GSHE|FMI|GSHE structure as shown in Fig. 3(c). The left GSHE acts as a spin injector and the right GSHE acts as a spin detector. Spins injected by the left GSHE via spin Hall effect, are transmitted through FMI via magnon diffusion, and detected by the right GSHE via inverse spin Hall effect. We have modularly attached the spin circuit for GSHE^20^ with the spin circuits for bulk magnon transport in FMI and FMI|NM interface, using standard circuit rules. We assumed same dimensions for injector and detector GSHE and simulated Pt|YIG|Pt structure using SPICE solver to observe the ratio of ISHE charge voltage per unit length of the detector GSHE to the charge current flowing in the injector GSHE (which we call *R*_*ISHE*_). Our simulation considers the spin diffusion along the extended region of YIG. For simulation, we assumed Pt thickness and width as 7 nm and 300 nm respectively. Pt resistivity, spin diffusion length, and spin Hall angle were taken from experimental report^5^. The cross-sectional area (*A*_*m*_) of magnon diffusion is assumed as 0.2 *μ*m × 100 *μ*m.

We compared our simulation results with the experiments by Cornelissen *et al.*^27^ to estimate spin circuit parameters for yttrium-iron-garnet (see Fig. 3(d)). Comparison with experiment yields: (1) magnon diffusion length (*λ*_*m*_) of ~10 *μ*m which agrees with the previously reported values at room temperature^27^,^48^, (2) magnon spin resistivity (*ρ*_*m*_) of ~10 *μ*Ω-cm, which is an order lower than previously reported value (~250 *μ*Ω-cm^24^,^27^), and (3) interface conductance per unit area for spins collinear to the magnetization direction (*g*_*s*_) of ~3.5 × 10^14^ S/m^2^, which is similar to the real part of the interface spin mixing conductance reported previously^21^,^33^. Details of comparison and estimation of parameters are provided as Supplementary Information. We noted the deviation of our extracted parameters with the prior reports and we provide detailed analysis of our spin funneling structure using different YIG parameters in the next section.

### Comparison with Theory

We connect our spin circuit for magnon transport with already existing spin circuit for GSHE^20^ using standard circuit rules to form GSHE|FMI|GSHE structure and with straightforward algebra (see Supplementary Information) we derive an analytical expression for the ratio of ISHE charge current density in the detector GSHE (*J*_*c*2_) to the applied charge current density in the injector GSHE (*J*_*c*1_), given by


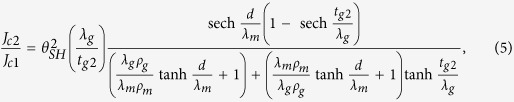


which has the exact form as of the standard result^23^ with the pure spin resistivity given by 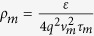, where *v*_*m*_ is the magnon velocity, *τ*_*m*_ is the magnon conserving scattering time, *q* is the electron charge, and *ε* is the boundary condition used in ref. 23. Note that interface resistance is not included in this formalism.

### Spin Transmission through YIG

Spin transmission through thin YIG is very high due to its large magnon diffusion length (~10 *μ*m). But spin injection into YIG from GSHE spin source is determined by spin resistances of GSHE and YIG layers and GSHE|YIG interface. The intrinsic spin current (*θ*_*SH*_*J*_*c*_) generated by spin Hall effect gets divided between the GSHE (spin) source resistance and FMI (spin) resistance (FMI bulk and FMI|GSHE interface resistance). High resistive GSHE layer is desired as a good spin source to inject spins into FMI.

## Detailed results

This section provides a discussion on the effects of different material and geometry parameters on spin funneling. We considered W(6)|YIG(4)|NM(20)|FM(2) structure for simulation (see Fig. 1(c)) where the numbers in the parentheses indicate the thicknesses in nm. Note that we performed a 2D simulation (to analyze 1D funneling) which misses spin diffusion from the width direction (perpendicular to the paper). We expect larger enhancement than reported here for 2D funneling structures, which are more difficult to analyze with proper 3D simulations, but should be no more difficult to fabricate.

### Dependence on g_s_ and ρ_m_

**Figure 4:**
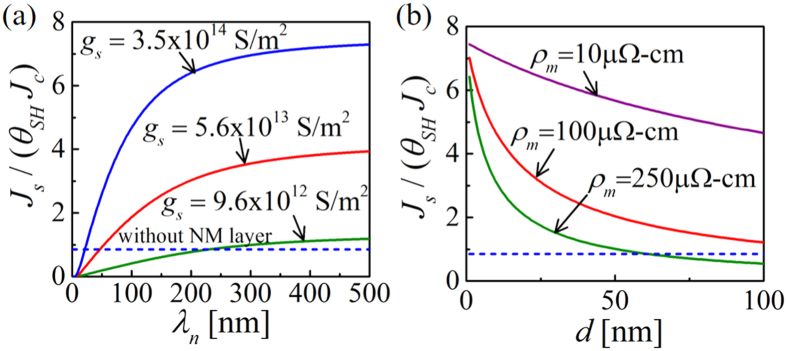
(**a**) *J*_*s*_/(*θ*_*SH*_*J*_*c*_) as a function of NM spin diffusion length (*λ*_*n*_) for different YIG|NM interface conductances: 9.6 × 10^12 ^S/m^2 ^ (ref. 24), 5.6 × 10^13 ^S/m^2 ^ (ref. 33), and 3.5 × 10^14 ^S/m^2^ (estimated by comparing our model with experiment^27^, see Supplementary Information). Magnon resistivity and diffusion lengths are assumed 10 *μ*Ω-cm and 10 *μ*m respectively for this simulation. (**b**) *J*_*s*_/(*θ*_*SH*_*J*_*c*_) as a function of YIG thickness for different magnon resistivity: 10 *μ*Ω-cm (estimated by comparing our model with experiment^27^, see Supplementary Information), 100 *μ*Ω-cm, and 250 *μ*Ω-cm^24^,^27^. The interface conductance and magnon diffusion length are assumed as 3.5 × 10^14 ^S/m^2^ and 10 *μ*m respectively for this simulation.

The effective spin Hall angle of the composite structure depends on the spin transmission efficiency of the PSC layer determined by the magnon resistivity (*ρ*_*m*_) and the FMI|NM interface conductance (*g*_*s*_). Fig. 4(a) shows the enhancement ratio (*J*_*s*_/(*θ*_*SH*_*J*_*c*_)) as a function of spin diffusion length (*λ*_*n*_) in the NM layer for different values of *g*_*s*_: 9.6 × 10^12^ S/m^2^ (reported in ref. 24), 5.6 × 10^13 ^S/m^2^ (reported in ref. 33), and 3.5 × 10^14^ S/m^2^ (estimated by fitting our model with experiments in ref. 27, which is similar to the real part of the interface spin mixing conductance of YIG^21^,^33^). *λ*_*n*_ is swept from 1 nm to 500 nm. Note that in our simulation we assume that YIG|Cu and YIG|W have same interface conductance. Maximum enhancement ratios are ~7 and ~4 for *g*_*s*_ being 3.5 × 10^14^ and 5.6 × 10^13^ S/m^2^ respectively. No enhancement in effective spin Hall angle is observed if the YIG|NM interface conductance is low (~9.6 × 10^12 ^S/m^2^).

**Figure 5:**
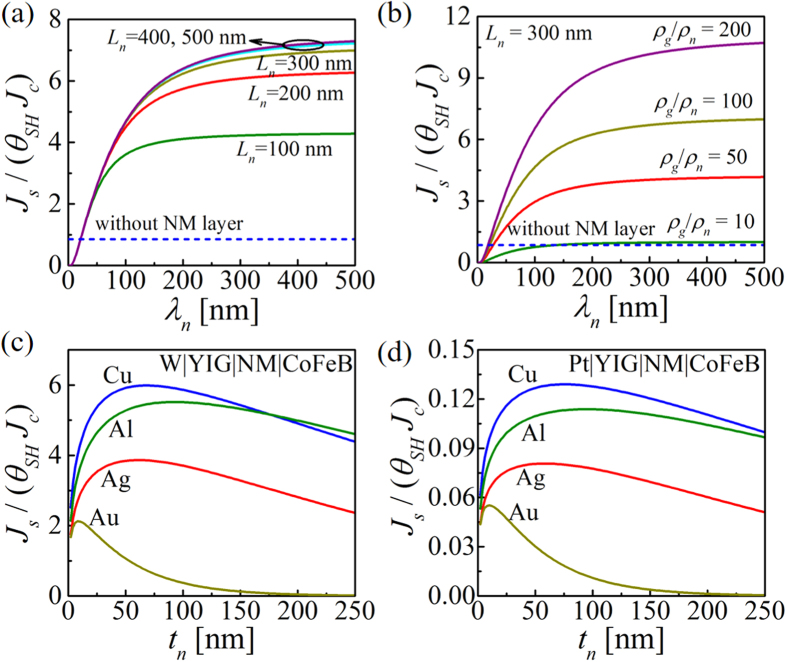
*J*_*s*_/(*θ*_*SH*_*J*_*c*_) as a function of NM spin diffusion length (*λ*_*n*_) for (**a**) different NM lengths (*L*_*n*_) and (**b**) for different GSHE to NM resistivity ratios (*ρ*_*g*_/*ρ*_*n*_). For shorter NM length, enhancement saturates at min(*λ*_*n*_, *L*_*n*_) and while for longer NM lengths saturation is determined by the resistance mismatch of GSHE and NM. Spin funneling is higher for *ρ*_*g*_ > *ρ*_*n*_. W|YIG|Cu|FM structure is simulated where FM is a perfect spin sink. To further evaluate these conclusions, we have used four different NM layers (Cu, Al, Ag, and Au) with two different GSHE spin sources **(c)** tungsten (W) and **(d)** platinum (Pt). We observe enhancement for W while degradation for Pt since W has resistivity an order higher than Pt. There exists a critical thickness of the NM layer related to *λ*_*n*_ for which spin funneling is maximum. For thick NM layer, spin funneling ability is Al>Cu>Ag>Au determined by their *λ*_*n*_. For very thin NM layer, Cu>Al since Cu has lower resistivity than Al and comparable spin diffusion lengths.

**Figure 6:**
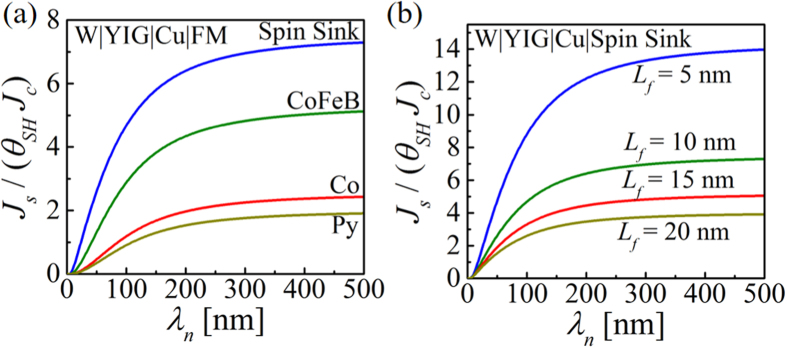
*J*_*s*_/(*θ*_*SH*_*J*_*c*_) as a function of NM spin diffusion length (*λ*_*n*_) for: (**a**) Different magnets: perfect spin sink, CoFeB, Co, and Py. Note that we are observing the maximum spin current absorbed by the FM. (**b**) Different FM lengths (*L*_*f*_). Enhancement doubles if we make the FM length half. This is because NM layer pins down the spin current injected into FM by spin funneling.

Fig. 4(b) shows the enhancement ratio as a function of the YIG layer thickness (*d*) for different values of magnon resistivities with *g*_*s*_ = 3.5 × 10^14 ^S/m^2^. For 4 nm thick YIG, the enhancement ratio are ~7 and ~5 for magnon resistivity 10 *μ*Ω-cm and 250 *μ*Ω-cm, respectively. The enhancement in effective spin Hall angle decreases faster with YIG thickness for high magnon resistivity case (~250 *μ*Ω-cm^24^,^27^), compared to the lower magnon resistivity case (~10 *μ*Ω-cm, estimated by comparing our model with experiment^27^). For our simulations in Fig. 5 and Fig. 6, we use *g*_*s*_ = 3.5 × 10^14^ S/m^2^, *λ*_*m*_ = 10 *μ*m, and *ρ*_*m*_ = 10 *μ*Ω-cm for 4 nm thick YIG.

## Dependence on λ_n_ and L_n_. 

Fig. 5(a) shows enhancement ratio as a function of NM spin diffusion length (*λ*_*n*_) for different NM lengths (*L*_*n*_). *λ*_*n*_ is swept from 1 nm to 500 nm for *L*_*n*_ = 100 to 500 nm. Copper is used as funnel layer in this simulation and FM is considered as a perfect spin sink. For short length (100 ~ 300 nm) of Cu layer, the enhancement ratio saturates at min(*λ*_*n*_, *L*_*n*_). This is because the spin funneling occurs due to the 2D diffusion of spins in the bulk NM and spins coming from a distance larger than spin diffusion length get lost in the bulk due to spin relaxation. For longer Cu lengths (≥400 nm), the saturation behavior is determined by the mismatch between GSHE source resistance and spin resistance of the NM layer, which is related to the resistivity ratio for GSHE and NM (i.e. *ρ*_*g*_/*ρ*_*n*_).

### Dependence on ρ_g_ and ρ_n_

To increase the spin funneling effect, the resistivity of the funnel layer has to be much lower than that of the spin source layer (GSHE) i.e. *ρ*_*g*_ ≫ *ρ*_*n*_, as shown in Fig. 5(b). Large enhancement is observed for a very low resistive funnel layer driven by a very high resistive GSHE spin source. The NM length is 300 nm for this simulation. To further evaluate the effect of resistivity mismatch between GSHE and NM layers and the effect of spin diffusion length of NM, we have performed simulations using realistic material parameters for different GSHE and NM, as shown in Fig. 5(c–d). In these simulations, we used CoFeB as the FM layer with its magnetization along the 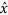-direction, orthogonal to the spin polarization direction (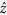-direction) in GSHE. Thus we are observing the maximum spin current density absorbed by the FM, which is determined by the real component of the interface spin mixing conductance (see Supplementary Information).

Fig. 5(c,d) show *J*_*s*_/(*θ*_*SH*_*J*_*c*_) as a function of NM layer thickness (*t*_*n*_) for two different spin sources: tungsten (W) and platinum (Pt) respectively. The resistivity of W (~200 *μ*Ω-cm) is about an order higher than that of Pt (~24 *μ*Ω-cm). Enhancement (≫1) caused by spin funneling is observed for the case where W is used as spin source (Fig. 5(c)) but degradation (<1) is observed for the case where Pt is used as spin source (Fig. 5(d)). This observation also has a similarity with the spin pumping experiment by Deorani and Yang^34^ where it is shown that an intermediate Cu layer shows enhancement of factor 2.2 for Ta|Cu|Py structure and enhancement <1 for Pt|Cu|Py structure.

### Different NM and Critical Funneling thickness

Four different NM materials are considered as funnel layer: copper (Cu), aluminum (Al), silver (Ag), and gold (Au) with resistivities of 2.08, 3.2, 5.5, and 5.2 *μ*Ω-cm respectively and spin diffusion lengths of 500, 600, 300, and 60 nm respectively (see Supplementary Information). For thicker NM layer, spin funneling ability in different NM materials used for simulation is in the following order: Al>Cu>Ag>Au mostly determined by their spin diffusion length. But for very thin NM layer compared to the spin diffusion length, low resistivity determines high spin funneling. Thus thin Cu layer shows better funneling than thin Al layer since resistivity of Cu is lower than Al. The observation that insertion of Cu as intermediate layer have better impact than Ag has similarity with the spin pumping experiment by Wang *et al.*^37^. There exists a critical thickness of the NM layer for which the spin funneling is maximum, which is proportional to *λ*_*n*_.

### Applicable for Low Spin Resistive Load

Spin funneling is a mechanism to enhance spin injection into low spin resistive load like FM. This is because NM layer collects spins from spin source which diffuses towards the lowest spin resistive path and FM acts as a spin sink. Spin funneling will not enhance spin injection into a load which is higher spin resistive than the funnel layer (e.g. semiconductors). Fig. 6(a) shows *J*_*s*_/(*θ*_*SH*_*J*_*c*_) as a function of NM spin diffusion length for different ferromagnets in W|YIG|Cu|FM structure. The blue curve shows the ideal spin sink case where other ferromagnets e.g. CoFeB, Co, Py shows lower enhancement based on the real part of the interface spin mixing conductance, as we are observing the maximum spin current absorbed by the FM. The interface spin mixing conductance for different FMs used for simulation are provided as the Supplementary Information.

### Larger Enhancement for Smaller FM

Enhancement ratio is higher for FM with smaller length as shown in Fig. 6(b) and the enhancement doubles if we make the FM length half. This is because for a fixed longitudinal charge current density in GSHE, the transverse spin current density (*J*_*s*_) injected into the FM is the spin current injected into (*I*_*s*_) per unit area i.e. *J*_*s*_ = *I*_*s*_/(*wL*_*f*_). When GSHE is directly driving a FM, *I*_*s*_ decreases proportional to the FM length. Thus Eq. (1) is independent of *L*_*f*_. But in the presence of spin funneling, *I*_*s*_ is kept fixed by the NM parameters. Hence *J*_*s*_ doubles if we make *L*_*f*_ half which in turn we observe in the enhancement ratio *J*_*s*_/(*θ*_*SH*_*J*_*c*_) for fixed charge current density in GSHE. FM is perfect spin sink in this simulation. For both simulations in Fig. 6, NM length is 300 nm.

## Summary

We propose that composite structures can be designed with existing spin Hall materials so that the effective spin Hall angle is larger by an order of magnitude, lowering the switching current in the structure. Using our experimentally benchmarked models we show that an intermediate normal metal layer with low resistivity and large spin diffusion length can funnel spins from large area of the spin Hall materal into the small area of ferromagnet. We show that the approach is increasingly effective as magnets get smaller and should help overcome the well-known challenge of switching small and stable magnets. To avoid the current shunting by the low resistive NM layer, we utilize recently discovered phenomenon of pure spin conduction (PSC) via magnon diffusion in ferromagnetic insulators (FMI). We use a thin layer of FMI to have good spin transmission for our composite structure. We provide a spin circuit model for magnon diffusion in the bulk of FMI, and modify already existing FMI|NM interface module to include interface spin conductance for spins collinear to the magnetization direction of FMI. We compare our model with recent experiment^27^ to estimate the model parameters for YIG, which we use to simulate our composite structure. Similarity between magnon assisted spin transport in FMI and spin transport in NM opens up the possibility of direct use of FMI for spin funneling without an additional NM layer. Combining the spin circuit for magnon transport in FMI with existing GSHE module^20^ we reconstruct standard theory^23^ using circuit rules and straightforward algebra. The spin circuit model for PSC will serve as another tool in our multi-physics framework^40^, which enables evaluation of innovative spin based device concepts in a relatively straightforward manner.

## Additional Information

**How to cite this article**: Sayed, S. *et al.* Spin Funneling for Enhanced Spin Injection into Ferromagnets. *Sci. Rep.* **6**, 28868; doi: 10.1038/srep28868 (2016).

## Supplementary Material

Supplementary Information
