# Supplementary material for: Spin Funneling for Enhanced Spin Injection into Ferromagnets
Source: Sci Rep. 2016 Jul 4;6:28868. doi: 10.1038/srep28868 (PMC4931498; doi:10.1038/srep28868)
Supplement: Supplementary Information [file srep28868-s1.pdf]

Spin Funneling for Enhanced Spin Injection into Ferromagnets: Supplementary Information

Shehrin Sayed, Vinh Q. Diep, Kerem Yunus Camsari, and Supriyo Datta
School of Electrical and Computer Engineering,
Purdue University, West Lafayette, IN 47907, USA

I. DERIVATION OF EQ. (1)

This appendix provides the straightforward derivation of Eq.(1) *in the main manuscript using spin circuit model of GSHE material [1].*

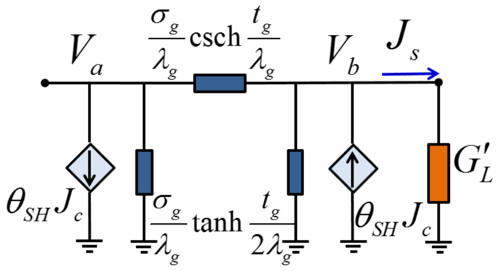

FIG. S1. Spin circuit representation for GSHE material [1] driving a spin load G'_L. Note that the inverse spin Hall effect is ignored in this derivation.

The spin circuit representation of giant spin Hall effect (GSHE) material driving a spin load (G'_L) is shown in FIG. S1. The equations at node a and b are given by:

$$\theta_{SH} J_c + V_a \frac{\sigma_g}{\lambda_g} \coth \frac{t_g}{\lambda_g} - V_b \frac{\sigma_g}{\lambda_g} \text{csch} \frac{t_g}{\lambda_g} = 0, \tag{I.1}$$

and

$$-\theta_{SH} J_c - V_a \frac{\sigma_g}{\lambda_g} \text{csch} \frac{t_g}{\lambda_g} + V_b \left(\frac{\sigma_g}{\lambda_g} \coth \frac{t_g}{\lambda_g} + G'_L \right) = 0, \tag{I.2}$$

where σ_g, λ_g, θ_{SH}, and t_g are conductivity, spin diffusion length, intrinsic spin Hall angle, and thickness of GSHE, respectively. From Eqs. (I.1) and (I.2), we have

$$V_a = -V_b \left(1 + \frac{G'_L}{\frac{\sigma_g}{\lambda_g} \tanh \frac{t_g}{2\lambda_g}} \right). \tag{I.3}$$

From Eq. (I.1) we get:

$$V_b = \theta_{SH} J_c \frac{\tanh \frac{t_g}{2\lambda_g}}{G'_L \coth \frac{t_g}{\lambda_g} + \frac{\sigma_g}{\lambda_g}}. \tag{I.4}$$

The spin current density injected into the spin load is given by

$$J_s = G'_L V_b = \theta_{SH} J_c \left(1 - \operatorname{sech}\frac{t_g}{\lambda_g}\right) \frac{G'_L}{G'_L + \frac{\sigma_g}{\lambda_g}\tanh\frac{t_g}{\lambda_g}}, \tag{I.5}$$

which gives Eq. (1) in the main manuscript.

II. SPIN CIRCUIT MODELS USED FOR SIMULATION

This appendix provides the description of GSHE, NM, and FM spin circuit models and provides corresponding material parameters.

A. GSHE model

GSHE model has a charge circuit along $\hat{x}$-direction and a three component spin circuit along $\hat{y}$ direction. The charge circuit parameters are:

$$I_g^c = \frac{w\theta_{SH}}{\rho_g}(V_3^s - V_4^s), \text{ and } G_g^c = \frac{wt_g}{\rho_g L_g}, \tag{II.1}$$

and the spin circuit parameters are:

$$I_g^s = \frac{w\theta_{SH}}{\rho_g}(V_1^c - V_2^c)[1\ 0\ 0]^T, \ G_g^{se} = G_{g0}\operatorname{csch}\frac{t_g}{\lambda_g} I_{3\times3}, \text{ and } G_g^{sh} = G_{g0}\tanh\frac{t_g}{2\lambda_g} I_{3\times3}, \tag{II.2}$$

where $G_{g0} = \dfrac{wL_g}{\rho_g\lambda_g}$ and $I_{3\times3}$ is the 3×3 identity matrix. The material parameters used in our simulations are given in the following table:

GSHE	λ_g [nm]	$\rho_g[\mu\Omega\text{-cm}]$	θ_{SH}
W [2]	2.1	200	0.33
Ta [3]	1.8	190	0.15
Pt [4]	1.2	24	0.07

Table 1: GSHE material parameters

B. NM model

The NM model is a four component (one charge and three spins) 1D transport model with the following parameters:

$$G_n^{se} = G_{n0}\begin{bmatrix} 1 & 0 & 0 & 0 \\ 0 & \frac{L_n}{\lambda_n}\operatorname{csch}\frac{L_n}{\lambda_n} & 0 & 0 \\ 0 & 0 & \frac{L_n}{\lambda_n}\operatorname{csch}\frac{L_n}{\lambda_n} & 0 \\ 0 & 0 & 0 & \frac{L_n}{\lambda_n}\operatorname{csch}\frac{L_n}{\lambda_n} \end{bmatrix}, \tag{II.3}$$

$$G_n^{sh} = G_{n0}\begin{bmatrix} 0 & 0 & 0 & 0 \\ 0 & \frac{L_n}{\lambda_n}\tanh\frac{L_n}{2\lambda_n} & 0 & 0 \\ 0 & 0 & \frac{L_n}{\lambda_n}\tanh\frac{L_n}{2\lambda_n} & 0 \\ 0 & 0 & 0 & \frac{L_n}{\lambda_n}\tanh\frac{L_n}{2\lambda_n} \end{bmatrix}, \tag{II.4}$$

where $G_{n0} = \dfrac{wt_n}{\rho_n L_n}$. The material parameters used in our simulations are given in the following table:

NM	λ_n [nm]	$\rho_n[\mu\Omega\text{-cm}]$
Cu [5–7]	300∼500	2.08
Al [6]	600	3.2
Ag [8, 9]	152, 300	5.5
Au [7]	60	5.2

Table 2: NM material parameters

C. FM model

The bulk FM model parameters are given by:

$$G_f^{se} = G_{f0}\begin{bmatrix} 1 & p & 0 & 0 \\ p & p^2 + \beta \operatorname{csch}\frac{t_f}{\lambda_f} & 0 & 0 \\ 0 & 0 & 0 & 0 \\ 0 & 0 & 0 & 0 \end{bmatrix}, \tag{II.5}$$

$$G_f^{sh} = G_{f0}\begin{bmatrix} 0 & 0 & 0 & 0 \\ 0 & \beta \tanh\frac{t_f}{2\lambda_f} & 0 & 0 \\ 0 & 0 & \frac{t_f}{\lambda_{ft}}\tanh\frac{t_f}{2\lambda_{ft}} & 0 \\ 0 & 0 & 0 & \frac{t_f}{\lambda_{ft}}\tanh\frac{t_f}{2\lambda_{ft}} \end{bmatrix}, \tag{II.6}$$

where $G_{f0} = \dfrac{wL_f}{\rho_f t_f}$ and $\beta = (1-p^2)\dfrac{t_f}{\lambda_f}$. The material parameters used in our simulations are given in the following table:

FM	p	λ_f [nm]	$\rho_f[\mu\Omega\text{-cm}]$
CoFeB	0.65 [10]	12 [12, 14]	168 [2]
Py	0.49 [11]	5.5 [12, 13]	23.1 [11]
Co	0.45 [10]	38 [14]	21 [14]

Table 3: FM material parameters

The transverse spin diffusion length (λ_{ft}) in FM is assumed to be very short.

D. FM|NM model

The FM|NM interface model parameters are given by:

$$G_{f/n}^{se} = [R]G_{fn0}\begin{bmatrix} 1 & p & 0 & 0 \\ p & 1 & 0 & 0 \\ 0 & 0 & 0 & 0 \\ 0 & 0 & 0 & 0 \end{bmatrix}[R]^\dagger, \text{ and } G_{f/n}^{sh} = [R]\begin{bmatrix} 0 & 0 & 0 & 0 \\ 0 & 0 & 0 & 0 \\ 0 & 0 & 2G_r^{\uparrow\downarrow} & 2G_i^{\uparrow\downarrow} \\ 0 & 0 & -2G_i^{\uparrow\downarrow} & 2G_r^{\uparrow\downarrow} \end{bmatrix}[R]^\dagger, \tag{II.7}$$

where $G_{fn0} = \dfrac{q^2}{h}\dfrac{k_F w}{\pi}$ is interface conductance and $[R]$ is a rotational matrix. The interface parameters used in our simulations are given in the following table:

FM	k_F [$\mathring{A}^{-1}$]	$G^{\uparrow\downarrow}_{r,(FM\|Cu)}$ [$\times 10^{15}$ Ω^{-1} m^{-2}]
CoFeB	1.04 [15, 16]	2.6 [17]
Py	1.05 [15, 16]	0.39 [13]
Co	0.96 [15, 16]	0.55 [18]

Table 4: FM|NM interface parameters

The imaginary component of the interface mixing conductance $G_i^{\uparrow\downarrow}$ is assumed to be two orders lower than its real part ($G_r^{\uparrow\downarrow}$). Note that in our simulation, we set the magnetization direction orthogonal to the injected spin polarization direction to observe the maximum absorbed spin current density by the FM. Thus only parameter actually affected the simulation is the real part of the interface spin mixing conductance ($G_r^{\uparrow\downarrow}$). Moreover, for spin sink simulations, we used ground boundary condition at the spin terminal instead of using bulk FM and FM|NM interface modules. This corresponds to $G_r^{\uparrow\downarrow} \to \infty$.

III. SPIN CIRCUIT FOR MAGNON TRANSPORT IN FMI

This appendix provides the formal derivation of the spin circuit model for 1D magnon diffusion in the bulk of ferromagnetic insulator. We compare our model with experiment [19] to estimate circuit parameters for YIG.

A. Model for bulk magnon diffusion in FMI

We start from the 1D magnon spin diffusion equation [21, 24] given by

$$\frac{d^2\mu_m}{dx^2} = \frac{\mu_m}{\lambda_m^2}, \tag{III.1}$$

where μ_m is the chemical potential for magnon accumulation [24] and λ_m is magnon diffusion length. Boundary conditions used at both interfaces are $\mu_m(x=0) = q\ V_1^s$ and $\mu_m(x=d) = q\ V_2^s$, where V_1^s and V_2^s are the external spin voltages at the left and right interfaces of FMI excluding the drop across the interface resistance. The excess drop across the interface will be modeled as a separate interface module, as mentioned in the Section III of the main manuscript. Under this boundary condition, the solution of Eq. (III.1) is given by

$$\mu_m(x) = \frac{V_2^s \sinh\left(\dfrac{x}{\lambda_m}\right) + V_1^s \sinh\left(\dfrac{d-x}{\lambda_m}\right)}{\sinh\left(\dfrac{d}{\lambda_m}\right)}. \tag{III.2}$$

The magnon assisted spin current density is given by

$$j_m = -\frac{\sigma_m}{q}\frac{d\mu_m}{dx}, \tag{III.3}$$

which is same as Ref. 21 and Ref. 24, except here it is written in A/m^2 unit. Here σ_m is the magnon pure spin conductivity given by [24]

$$\sigma_m = 2q^2 \bar{D}_m \frac{d\delta n_m}{d\mu_m}, \tag{III.4}$$

where q is the electron charge, δn_m is the non-equilibrium magnon density, $\bar{D}_m = v_m^2\tau_m$ is the magnon diffusion constant, v_m is the magnon velocity, τ_m is the magnon conserving scattering time.

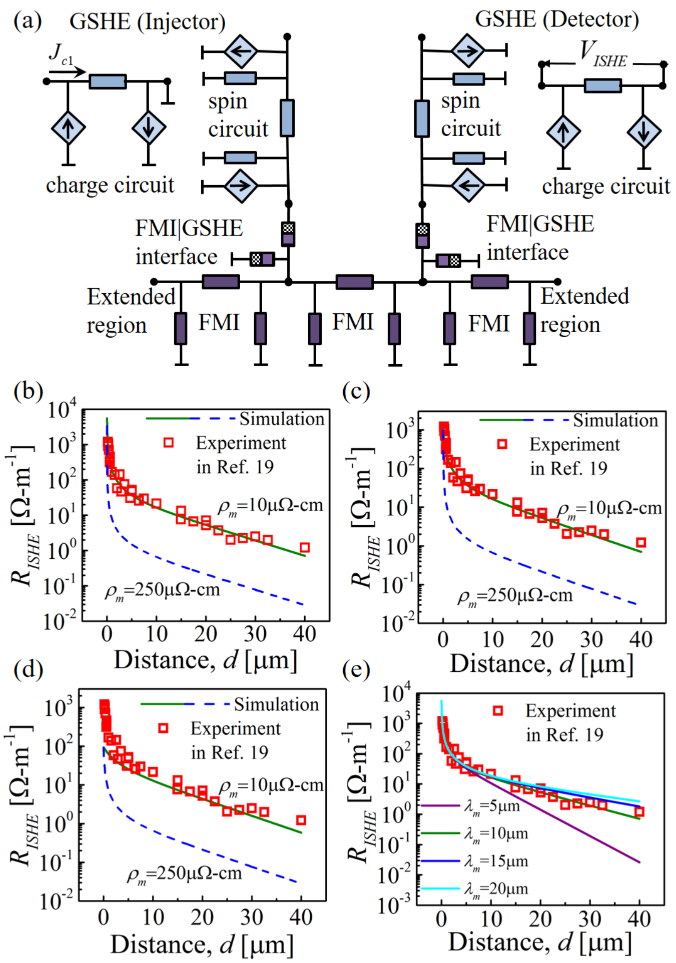

FIG. S2. **(a)** Spin circuit representation of the structure in Fig. 3(c) in the main manuscript. We use spin circuits for bulk FMI (see Fig. 3(a) of the main manuscript), FMI|NM interface (see Fig. 3(b) of the main manuscript), and spin circuit for GSHE [1]. Left most and right most FMI block captures the spin diffusion in the extended region of FMI sample. The spin circuit description can be found in Section II of this supplementary information (also see Fig. 2(c) in the main manuscript). R_{ISHE} is the ratio of inverse spin Hall voltage per unit length on the detector GSHE to the charge current flowing in the injector GSHE as shown in the structure Fig. 3(c) in the main manuscript. We observe simulation result for R_{ISHE} as a function of distance between two GSHE layers and compare with experiments in Ref. 19. Comparison is shown for different YIG parameters: YIG|NM interface conductance (g_s), magnon resistivity (ρ_m), and magnon diffusion length (λ_m). **(b)** $g_s = 3.5{\times}10^{14}$ S/m^2, $\lambda_m = 10$ μm, and $\rho_m = 10$ and 250 $\mu\Omega$-cm. **(c)** $g_s = 5.6{\times}10^{13}$ S/m^2, $\lambda_m = 10$ μm, and $\rho_m = 10$ and 250 $\mu\Omega$-cm. **(d)** $g_s = 9.6{\times}10^{12}$ S/m^2, $\lambda_m = 10$ μm, and $\rho_m = 10$ and 250 $\mu\Omega$-cm. **(e)** $g_s = 3.5{\times}10^{14}$ S/m^2, $\lambda_m = 5$, 10, 15, and 20 μm, and $\rho_m = 10$ $\mu\Omega$-cm. Good match with experiment is observed for $g_s = 3.5{\times}10^{14}$ $\mu\Omega$-cm, $\lambda_m = 10$ μm, and $\rho_m = 10$ $\mu\Omega$-cm.

The magnon current entering from $x = 0$ and $x = d$ are given by

$$I^s_{m,1} = \int\int j_m|_{x=0}\, dydz \;\; \text{and} \;\; I^s_{m,2} = -\int\int j_m|_{x=d}\, dydz, \tag{III.5}$$

noting that positive current means current going into the surface. Note that the spin polarization is collinear with the magnetization direction of FMI. Combining Eqs. (III.2), (III.3), and (III.5) yields the conductance

matrix for magnon transport in the bulk of FMI as

$$\begin{pmatrix} I^s_{m,1} \\ I^s_{m,2} \end{pmatrix} = \frac{\sigma_m A}{\lambda_m} \begin{bmatrix} \coth\left(\frac{d}{\lambda_m}\right) & -\operatorname{csch}\left(\frac{d}{\lambda_m}\right) \\ -\operatorname{csch}\left(\frac{d}{\lambda_m}\right) & \coth\left(\frac{d}{\lambda_m}\right) \end{bmatrix} \begin{pmatrix} V^s_1 \\ V^s_2 \end{pmatrix}. \tag{III.6}$$

The conductance matrix in Eq. (III.6) is translated into the spin circuit representation shown in FIG.3(a) in the main manuscript with the series and shunt conductances given by Eq. (3) in the main manuscript.

B. Estimation of Spin Circuit Parameters

We have simulated Pt|YIG|Pt structure in Fig. 3(c) in the main manuscript using our spin circuit models (see FIG. S2(a)). We connected the spin circuit for the corresponding layer or interface using standard circuit rules and simulated the structure with standard SPICE solver using various parameters values for: interface conductance (g_s), magnon resistivity (ρ_m), and magnon diffusion length (λ_m). The cross-sectional area (A_m) for magnon diffusion is the thickness of YIG (200 nm) times the length of the Pt layer (100 μm). The spin diffusion in the extended regions of FMI on both left and right sides of the injector and detector GSHEs respectively, are taken into account by two additional bulk FMI blocks. We assumed that the length of the extended FMI region is much longer than the magnon diffusion length. Dimensions of the injector and detector GSHEs are same with thickness and width 7 nm and 300 nm respectively. The parameters for platinum [4] used here are: $\rho_g = 24$ $\mu\Omega$-cm, $\lambda_g = 1.2$ nm, and $\theta_{SH} = 0.07$. We look at the inverse spin Hall voltage per unit length at the detector GSHE to charge current in the injector GSHE ratio as a function of distance (d) between two GSHE contacts and compare with the experiments in Ref. 19. The comparison is shown in FIG. S2(b)-(e).

FIG. S2 (b), (c), and (d) shows simulation for three different interface conductance: 3.5×10^{14} S/m^2 (similar to the real part of the interface spin mixing conductance [22, 23]), 5.6×10^{13} S/m^2 [23], and 9.6×10^{12} S/m^2 [24] respectively. Each of the plot shows two simulations for two different values of magnon resistivity: 10 $\mu\Omega$-cm and 250 $\mu\Omega$-cm and magnon diffusion length was kept at 10 μm for all three plots. The interface conductance affects the simulation results for devices with smaller distance between two GSHE contacts. The change in magnon resistivity causes a constant shift for devices with large distance between two GSHE contacts. The change in magnon diffusion length changes the slope of the simulation results for devices with large distance between two GSHE contacts (see Fig. S2(d)). Our best fitting yields: $g_s = 3.5\times10^{14}$ S/m^2, $\rho_m = 10$ $\mu\Omega$-cm, and $\lambda_m = 10$ μm, which is the case shown in the green curve of FIG. S2(a).

IV. DERIVATION OF EQ. (5)

This appendix provides the derivation for Eq. (5) *in the main manuscript.*

We represent a GSHE|FMI|GSHE structure by attaching the spin circuit for magnon diffusion introduced in Section III of the main manuscript with the spin circuit of GSHE reported previously [1] using standard circuit rules (see FIG. S3). We ignore the interface conductance in this formalism (i.e. $g_s \to \infty$). We assumed thick GSHE as injector ($t_{g1} \gg \lambda_g$), same as the assumption in Ref. 21. For the detector GSHE, node c of spin circuit and the charge terminals in charge circuit are open circuited. We ignored the ISHE sources and SHE sources in the injector and detector GSHE circuit representations, respectively. Circuit parameters in FIG. S3 are given in Eq. (3) in the main manuscript and "GSHE module" subsection in Section II of this supplementary document. Note that the conductances are written in per unit area. Nodal equations at nodes a, b, and c are given by

$$\left(\frac{\coth\frac{d}{\lambda_m}}{\rho_m\lambda_m} + \frac{1}{\rho_g\lambda_g}\right) V_a - \frac{\operatorname{csch}\frac{d}{\lambda_m}}{\rho_m\lambda_m} V_b = \theta_{SH} J_{c1}, \tag{IV.1}$$

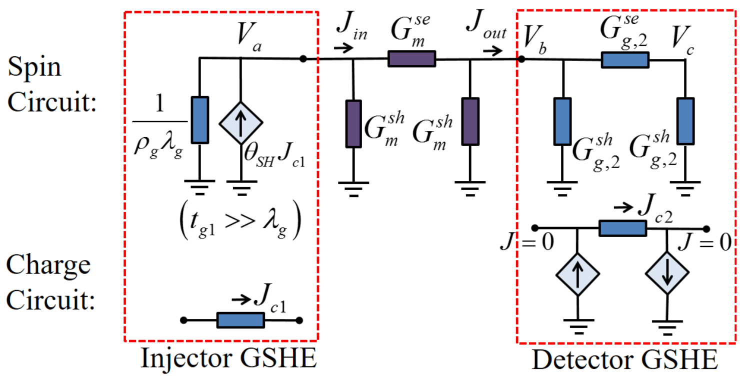

FIG. S3. Spin circuit representation of a GSHE|FMI|GSHE structure similar to Fig. 3(c) in the main manuscript. Each material is represented by corresponding spin circuit and they are connected together using standard circuit rules for charge and spin. Injector GSHE is assumed to be thick ($t_{g1} \gg \lambda_g$) and ISHE sources in injector GSHE and SHE sources in detector GSHE are neglected.

$$-\frac{\operatorname{csch}\frac{d}{\lambda_m}}{\rho_m\lambda_m}V_a+\left(\frac{\coth\frac{d}{\lambda_m}}{\rho_m\lambda_m}+\frac{\coth\frac{t_{g2}}{\lambda_g}}{\rho_g\lambda_g}\right)V_b-\frac{\operatorname{csch}\frac{t_{g2}}{\lambda_g}}{\rho_g\lambda_g}V_c=0, \tag{IV.2}$$

and

$$V_b=\cosh\frac{t_{g2}}{\lambda_g}V_c. \tag{IV.3}$$

From Eqs. (IV.1), (IV.2), and (IV.3), we get

$$V_b-V_c=\frac{\left(1-\operatorname{sech}\frac{t_{g2}}{\lambda_g}\right)\theta_{SH}J_{c1}}{\frac{\sinh\frac{d}{\lambda_m}}{\rho_m\lambda_m}+\frac{\cosh\frac{d}{\lambda_m}}{\rho_g\lambda_g}+\frac{\rho_m\lambda_m}{\rho_g\lambda_g}\left(\frac{\cosh\frac{d}{\lambda_m}}{\rho_m\lambda_m}+\frac{\sinh\frac{d}{\lambda_m}}{\rho_g\lambda_g}\right)\tanh\frac{t_{g2}}{\lambda_g}}. \tag{IV.4}$$

From charge circuit of detector GSHE, we have

$$J_{c2}=\frac{\theta_{SH}}{\rho_g t_{g2}}\left(V_b-V_c\right). \tag{IV.5}$$

Combining Eqs. (IV.4) and (IV.5) yields the expression in Eq. (5) in the main manuscript, which is the standard result in Ref. 21 noting the pure spin resistivity as $\rho_m=\frac{\epsilon}{4q^2v_m^2\tau_m}$.

[1] Hong, S., Sayed, S., & Datta, S. Spin circuit representation for the spin Hall effect. *Nanotechnology, IEEE Transactions on* **15**, 225-236 (2016).
[2] Pai, C. -F. *et al.* Spin transfer torque devices utilizing the giant spin Hall effect of tungsten. *Appl. Phys. Lett.* **101**, 122404 (2012).

[3] Liu, L. *et al.* Spin-Torque Switching with the Giant Spin Hall Effect of Tantalum. *Science* **336**, 555-558 (2012).

[4] Liu, L., Lee, O. J., Gudmundsen, T. J., Ralph, D. C. & Buhrman, R. A. Current-Induced Switching of Perpendicularly Magnetized Magnetic Layers Using Spin Torque from the Spin Hall Effect. *Phys. Rev. Lett.* **109**, 096602 (2012).

[5] Jedema, F. J., Filip, A. T. & van Wees, B. J. Electrical spin injection and accumulation at room temperature in an all-metal mesoscopic spin valve. *Nature* **410**, 345-348 (2001).

[6] Jedema, F. J., Nijboer, M. S., Filip, A. T. & van Wees, B. J. Spin injection and spin accumulation in all-metal mesoscopic spin valves. *Phys. Rev. B* **67**, 085319 (2003).

[7] Kimura, T., Hamrle, J. & Otani, Y. Estimation of spin-diffusion length from the magnitude of spin-current absorption: Multiterminal ferromagnetic/nonferromagnetic hybrid structures. *Phys. Rev. B* **72**, 014461 (2005).

[8] Godfrey, R. & Johnson, M. Spin Injection in Mesoscopic Silver Wires: Experimental Test of Resistance Mismatch. *Phys. Rev. Lett.*, **96**, 136601 (2006).

[9] Fukuma, Y. *et al.* Giant enhancement of spin accumulation and long-distance spin precession in metallic lateral spin valves. *Nat. Materials* **10**, 527–531 (2011).

[10] Huang, S. X., Chen, T. Y. & Chien, C. L. Spin polarization of amorphous CoFeB determined by point-contact Andreev reflection. *Appl. Phys. Lett.* **92**, 242509 (2008).

[11] Kimura, T., Sato, T. & Otani, Y. Temperature Evolution of Spin Relaxation in a NiFe/Cu Lateral Spin Valve. *Phys. Rev. Lett.* **100**, 066602 (2008).

[12] Taniguchi, T., Yakata, S., Imamura, H. & Ando, Y. Determination of Penetration Depth of Transverse Spin Current in Ferromagnetic Metals by Spin Pumping. *Appl. Phys. Express* **1**, 031302 (2008).

[13] Bauer, G. E. W., Tserkovnyak, Y., Hernando, D. H. & Brataas, A. Universal angular magnetoresistance and spin torque in ferromagnetic/normal metal hybrids. *Phys. Rev. B* **67**, 094421 (2003).

[14] Taniguchi, T., Yakata, S., Imamura, H. & Ando, Y. Penetration Depth of Transverse Spin Current in Ferromagnetic Metals. *Magnetics, IEEE Transactions on* **44**, 2636-2639 (2008).

[15] Petrovykh, D. Y. *et al.* Spin-dependent band structure, Fermi surface, and carrier lifetime of permalloy. *Appl. Phys. Lett.* **73**, 3459-3461 (1998).

[16] Li, Y. & Bailey, W. E. Wave-Number-Dependent Gilbert damping in metallic ferromagnets. *Phys. Rev. Lett.* **116**, 117602 (2016).

[17] Boone, C. T., Nembach, H. T., Shaw, J. M. & Silva, T. J. Spin transport parameters in metallic multilayers determined by ferromagnetic resonance measurements of spin-pumping. *J. of Appl. Phys.*, **113**, 153906 (2013).

[18] Xia, K., Kelly, P. J., Bauer, G. E. W., Brataas, A. & Turek, I. Spin torques in ferromagnetic/normal-metal structures. *Phys. Rev. B* **65**, 220401(R) (2002).

[19] Cornelissen, L. J., Liu, J., Duine, R. A., Youssef, J. B. & van Wees, B. J. Long-distance transport of magnon spin information in a magnetic insulator at room temperature. *Nat. Phys.* **11**, 1022-1026 (2015).

[20] Cornelissen, L. J., Peters, K. J. H., Duine, R. A., Bauer, G. E. W. & van Wees, B. J. Magnon spin transport driven by the magnon chemical potential in a magnetic insulator. *arXiv:1604.03706v1 [cond-mat.mes-hall]* (2016).

[21] Zhang, S. S. -L. & Zhang, S. Magnon Mediated Electric Current Drag Across a Ferromagnetic Insulator Layer. *Phys. Rev. Lett.* **109**, 096603 (2012).

[22] Chen, Y. -T. *et al.* Theory of spin Hall magnetoresistance. *Phys. Rev. B* **87**, 144411 (2013).

[23] Flipse, J. *et al.* Observation of the Spin Peltier Effect for Magnetic Insulators. *Phys. Rev. Lett.* **113**, 027601 (2014).

[24] Cornelissen, L. J., Peters, K. J. H., Duine, R. A., Bauer, G. E. W. & van Wees, B. J. Magnon spin transport driven by the magnon chemical potential in a magnetic insulator. *arXiv:1604.03706v1 [cond-mat.mes-hall]* (2016).
